# Diamond Open Access and Fair Science: Driving Global Impact at *Food Technology and Biotechnology*

**Published:** 2026-09-15

**Authors:** Božidar Šantek

**Affiliations:** Food Technology and Biotechnology journal, Faculty of Food Technology and Biotechnology University of Zagreb, Pierottijeva 6, 10 000 Zagreb, Croatia

## Dear readers,

It is a great honour to address you as the new Editor-in-Chief of the journal Food Technology and Biotechnology (FTB), a diamond open access journal published by the University of Zagreb Faculty of Food Technology and Biotechnology. I would like to begin by expressing my sincere gratitude to my predecessors, Prof. Pavao Mildner and Prof. Vladimir Mrša, for their dedicated work and many achievements. Their efforts secured the journal's international visibility and its inclusion in various scientific journal databases, beginning in 1999 when FTB was first indexed in the *Current Contents*.

I would also like to extend my special thanks to the journal’s Editor Iva Grabarić Andonovski, Linguistic Editor Zrinka Pongrac Habdija and Metrological Editor Prof. Zorana Grabarić, who have worked diligently for many years to develop and improve our journal. I sincerely hope they will continue to do so in the years ahead.

Four Deputy Editors from our Faculty, Prof. Ivona Elez Garofulić, Prof. Draženka Komes, Assoc. Prof. Blaženka Kos and Prof. Ivana Rumbak, are also involved in the journal's operations to help accelerate and improve the peer review and publication processes.

We would like to thank all previous members of the Editorial and Advisory Boards for their valuable contributions and welcome our new international Editorial Board, comprising 27 members, and our new Advisory Board, comprising 10 members. Both Boards are elected for a three-year term, with the possibility of two consecutive mandates, in accordance with the journal’s bye-laws (*1*). We hope that this will bring new momentum to the journal and help us reach the goal of becoming one of the leading diamond OA journals in the fields covered by FTB.

It is also essential to highlight the invaluable contribution of the Field editors and reviewers, without whose diligent work the publication of our journal would not be possible. I therefore wish to express my deep gratitude to them as well. I look forward to working together to further improve and expedite the review and publication process.

FTB will continue to implement best practices in accordance with the Diamond Open Access Standard (*2**,**3*). We will also continue to uphold the principles of accessibility, equity, diversity, and inclusion, as well as our commitment to environmental protection. As a signatory of the UN SDG Publishers Compact initiative (*4*) and an endorser of the SAGER initiative (*5*), we are committed to incorporating these principles into the journal’s workflows and policies. Therefore, we are encouraging authors to assign SDGs to their papers to promote the goals and add value to their paper. We also expect authors to follow the SAGER guidelines for a more systematic approach to the reporting of sex and gender in their research.

We have also introduced two new requirements in the submission system asking authors to indicate whether the manuscript was prepared with the assistance of an AI tool and whether a preprint version is available. This information helps us monitor if the use of AI tools and links to preprints are appropriately disclosed in the manuscript. It also helps us interpret and refine the results of Similarity Check. We are also working on expanding the instructions for review papers. Following the latest practices, we are introducing a methodology section in reviews so that authors can strengthen the trustworthiness of their work, and this will soon be reflected in our Instructions to Authors.

Our latest news is that FTB is joining the ResearchGate community. Our application has been approved and we are now in the process of activating the subscription. Research­Gate's Journal Home is a platform that offers new ways of en­gaging with authors and readers, and increases the visibility and reach of open access journals. 

The newly released Journal Citation Reports (*6*) shows that the Impact Factor (IF) of FTB has increased to 2.9, compared with 2.5 in 2025. The journal remains in Q3 in the fields of *Biotechnology & Applied Microbiology* and *Food Science & Technology*. At the same time, its Q2 ranking in Scopus, together with the increase of its IF, provides encouragement to continue our dedicated efforts to uphold high standards of scientific excellence and research integrity, as well as to further improve the quality of the published work and strengthen the journal's international visibility.

In conclusion, I would like to thank everyone who contributes, directly or indirectly, to the continued development of FTB and to its long-standing tradition of diamond open access publishing. I hope that, together, we will continue to drive the journal’s progress and enhance its visibility within the international scientific community. I am confident that, through our shared commitment and efforts, we can achieve these goals, even though the path ahead will undoubtedly present challenges.

Editor-in-Chief:


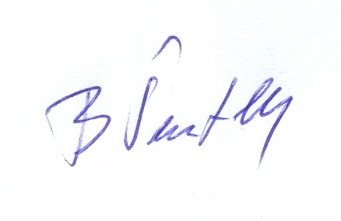


Prof. Dr. Sc. Božidar Šantek
